# Dental Fees

**Published:** 1909-12-15

**Authors:** 


### DENTAL FEES.

BY BROTHER BILL.

I have been in New York a week, and have enjoyed it immensely. I'm not going to write about the city now as I'm so full of some entirely new material I've stumbled onto for a dental society paper that I must tell you about it. You know I'm down for a paper on the subject of "Dental Fees." I needed some particular information for it, and while my friends were showing me about the city they led me onto just what I was looking for.

I suppose I've talked to as many dentists on the subject of fees as any one. It's a hobby of mine. I know just what I went through during the years when my fees yielded me no profits, and I know how much better my practice is *in every way* and how much more I enjoy life ever since I began making and saving money.

There was a time when I looked with a sort of sneaking admiration, which I wouldn't have admitted for the world, on the dentist who ran an advertising practice, and had a few diamonds scattered around his person, finely furnished offices, and was living on the fat of the land. I knew I was more ethical than he, that is professionally more holy, but I knew that I couldn't pay for diamonds like those; I couldn't support the fine offices; and I wasn't living so well either.

I don't feel that sneaking admiration any more. I've got a diamond or two, and a good horse or two, and as good an office as I want; and you couldn't drag me into his style of practice. It's having he money that makes the difference. It's easier to be ethical when you're making money than when you aren't. So I'm very glad I'm down for the subject of "Dental Fees," because I want to help men to be happily ethical by becoming ethically prosperous.

Whenever I talk to a dentist who is getting low fees, about raising his prices, he says, "I can't; Dr Blank works for these fees, and I cannot charge more and hold my practice." That's the answer I always get. And I've been searching for just the right reply to it. I've usually called a dentist's attention to the fact that the stores in his own town are not conducted on such a plan, but that they differ in prices and still do business. Now I've come across perhaps the most marked example of this in the country, and I'm going to use it in my paper.

Among the sights my friends showed me was the Mills Hotel on Seventh Avenue. It is conducted for the benefit of men who are financially reduced to the lowest point where they can live respectably. The building is equal to a fine office building, and towers many stories in the air. Here an outside room can be had for the sum of forty cents a day or an inside room for thirty cents. We hired a room to look it over, because I wanted the exact facts.

The forty-cent room faced on the street and had a large window. It seemed scrupulously clean and contained a narrow spring bed, furnished with clean bed clothing, a comfortable chair, a steel safe in the corner, where anything of value might be stored, and a steam radiator. There was a transom over the door to insure ventilation. While the room was small, it was clean and comfortable.

The dining-room attracted my special attention, and at my request my friends consented to eat there. It is very large and light, with a modern tile floor, the walls wainscoted in marble, and with good pictures as decorations. I send you herewith some pictures postals with explanations written on them.

It is enough to say here that the dinner was excellent. The portion was large; the food was of good quality and well cooked. The only drawback was that the service was rather noisy. The bill for an ample meal comprising soup, fried oysters, two vegetables, dessert and coffee was twenty-five cents. I was completely astounded that such service was possible at such figures. A little questioning of the people in charge developed the fact that the chef was of known skill, that all supplies were of excellent quality and that the help was paid as well as in other hotels thereabouts.

As I sat looking out of the window, I saw directly across the street, the Hotel York. It came to me as a surprise that another hotel should be so close, and that it should be able to thrive under such competition. Yet it looked prosperous; well-dressed people were coming and going; fine hangings were at the windows, and if it were about to fall into the hands of the sheriff, no outward sign of impending ruin was visible. It came over me like a flash, "If hotels can prosper so close together and at such entirely different scales of prices, why cannot dentists do the same" Why must a dentist reduce his fees or keep them down just because a dentist near him gets poor fees?

This struck me all the more forcibly because I know what nine dentists out of ten would have done as a means of meeting such competition. If a dentist opened a well fitted up office across the street and offered gold fillings for a dollar, most dentists would probably put the price of gold fillings in their offices down to a dollar, and maybe to ninety cents. They'd think "A gold filling is a gold filling, whether it is done in one office or another, and if that chap across the street offers them for a dollar, *of course* my only resource is to beat him to it."

I made up my mind immediately to see the man in charge of the other hotel. I felt sure the facts I got from him would supply the best material for my paper. So, having learned all I needed to about the Mills Hotel, we went across the street to see if this hotel man had been compelled to cut his prices, or if he was about to fail, or if he wanted to sell out because of the prices quoted by his neighbor.

A little inquiry brought the manager. Certainly he looked prosperous enough. No sign of the man ground down showed about him. And the office of the hotel looked busy and well-to-do. I told him my story and just what information I wanted. Then I proceeded to ask him the questions uppermost in my mind. I wanted to know what effect the Mills Hotel had on his business, whether he had reduced his prices, whether he had lost patrons because of it, and if so, what his remedy was. He was very courteous and answered each question promptly. His rates were from $1 to $3 a day for rooms alone. Meals were *a la carte.* No, he had not lost any patrons because of the Mills Hotel, so far as he was able to learn. He had not reduced his prices and did not see any reason for doing so. Business went along just as when the Mills Hotel was not there. Then he threw a flood of light on intelligent business methods, by a few sentences of explanation.

"You see people are not all of a class, and don't all want the same kind of service. There are many people who cannot afford to come here to eat or sleep and for such people the restaurants about town can afford accommodation at any price desired. For the man who is hard up, the Mills Hotel is a blessing. Such a man couldn't come here anyway. But the man who can afford good service wants it and is willing to pay for it. I notice that people generally want just as good service as they can afford. The man from the Mills Hotel, who gets on his feet, goes to a medium priced hotel and then to a house like this. Later, with increasing prosperity, he may move to a still higher priced house. Its attention and service and agreeable surroundings he wants and he's willing to pay. That's what keeps us all going. If you want further proof of this go up into Times Square and note the restaurants on the opposite sides. They'll be a lesson to you."

We thanked him, and withdrew, I with my mind too full of what he had said, to want to talk. There was the keynote, we as dentists so often miss "*People are of different classes. They want the best they can afford, and are willing to pay for it.*" When we get that firmly in our minds, the practice of dentistry will be a much straighter road to prosperity.

Next day we had lunch on the west side of Times Square, at one of the restaurants run by a firm famous all over the East. The room was beautifully fitted up, at a cost I was told, of over five thousand dollars. Certainly it was a room which a few years ago would have been considered a marvel. Everything seemed scrupulously clean. The food was of very good quality, indeed, and politely served by a neat waitress. The checks for lunch averaged thirty cents each.

My friends had now become interested in studying their own town, and that evening we went to the hotel for dinner. We entered through a pleasing portal and found ourselves in a beautiful room, which immediately set itself apart from the restaurants we had visited. So completely did this atmosphere envelop one, that he would have been surprised to have found charges there like those in the restaurants. Some oysters, a little fish, a cup of tea, and a piece of pie cost me a dollar without the tip. That's three times the price of the dinner at the Mills Hotel. There wasn't any more dinner and I don't know that it was any better food or better cooked. But as I sat listening to the orchestra and watching the well-dressed people come and go, I got the force of the hotel man's words "People like as good service as they can afford." And I know that if I lived here I would much rather pay the dollar for the same dinner, under these conditions, than eat at the Mills Hotel for thirty cents.

The restaurant below the hotel charges higher prices than the hotel. Inquiry showed that the average dinner check was about $2.00, not including wines, which in many cases carried it much higher.

The last restaurant visited was higher than the one just mentioned. Here it was no small task to get out with less than $3.00 for a respectable dinner for one, without anything stronger than coffee to drink.

What an illustration were these eating places of the hotel man's words of explanation. It is probably not more than 400 feet from the twenty-five cent places to the $4.00 place, and in that distance one passes a hotel and another restaurant. All are as busy as they care to be. From the crowd which daily passes these doors, each sifts out *by its service and fees*, the class of people who desire that service. Probably many in the twenty-five cent class look forward to the days when the dollar or two dollar class will not be out of their reach. And as soon as they can afford it, they will go to it. I've heard several persons express the hope that they might some time be able to afford the higher priced places.

That hotel manager furnished the material for my paper on "Dental Fees." I realize as never before that people want the best service they can afford as soon as they are educated to its advantages and pleasures. Each of these restaurants and hotels is really an institution leading the taste of the people upward. I never realized how I've been led along. The first time I went into the dining-room of a good hotel and saw the well-dressed people and sat among the beautiful flowers and heard the orchestra discourse sweet music, it was an event in my life—an educational event. It spoiled my taste for the place where the food is thrown out, helter-skelter. It made me want to get back to the better place; and it made me pay the bill with a smile.

There is no hotel man whose opportunities for educating the public to good services and good fees, excel the dentist's. The hotel man ministers to the senses only, but the dentist ministers to the appearance of the person, the well-being and the vigor of the whole body, the health of the stomach, the freedom from infectious diseases, and the duration of life. He has a hundred arguments where the hotel man has one. And he can make them just as winning as can these men, who in the face of keen competition are building up fortunes.

There's the substance of my paper. I hope it may not fall on deaf ears. There are just the same classes of people in our town that there are here. And they can be led through the same stages to the same ends.

For the dentist who sees nothing but price to dental work, the only way to fight competition may be to make lower prices. But for the dentist who can grasp the thought that each piece of dental work is something more than so much silver and gold put into a mouth and more than so much mere grinding and pounding, there is very much more.

Properly regarded, dentistry is mainly service. It consists in true perceptions of the patient's needs and skilful meeting of the requirements. Into this meeting the greater man will put so much of himself that his service becomes individual, a thing to be had only at his hands, to be appreciated as such, and to be willingly paid for on this plane. This is true now in thousands of cases.

These men render their services without bombast or self mention. They impress the patient as being too big to need such things. Their manner, their occupation in the work and the high degree of skill their service manifests, say without speech more than any words could say for them. I know such men whom I would pay fifty dollars, if necessary, to fill a tooth; and I know others whom I would rather hire to let it alone. That's a difference in men, in service; the difference in fees between them comes as naturally as the tail to a kite.

My friends have offered to show me the same sort of differences in other lines of service, and I may write you further. But I've got my text: "Educate your patients to good services at good fees."--*Digest.*

